# Problems paying medical bills and mental health symptoms post-Affordable Care Act

**DOI:** 10.3934/publichealth.2020023

**Published:** 2020-05-06

**Authors:** Jacqueline C Wiltshire, Kimberly R Enard, Edlin Garcia Colato, Barbara Langland Orban

**Affiliations:** 1 College of Public Health, University of South Florida, USA; 2 Department of Health Management and Policy, College for Public Health & Social Justice, Saint Louis University, USA

**Keywords:** problems paying medical bills, mental health, Affordable Care Act, insurance coverage

## Abstract

Healthcare affordability is a worry for many Americans. We examine whether the relationship between having problems paying medical bills and mental health problems changed as the Affordable Care Act (ACA) was implemented, which increased health insurance coverage. Data from the 2013–2016 Health Reform Monitoring Survey, a survey of Americans aged 18–64, were used. Using zero-inflated negative binomial regression, adjusted for predisposing, enabling, and need factors, we examined differences in days of mental health symptoms by problems paying medical bills (n = 85,430). From 2013 to 2016, the rates of uninsured and problems paying medical bills decreased from 15.1% to 9.0% and 22.0% to 18.6%, respectively. Having one or more days of mental health symptoms increased from 39.3% to 42.9%. Individuals who reported problems paying medical bills had more days of mental health symptoms (Beta = 0.133, p < 0.001) than those who did not have this problem. Insurance was not significantly associated with days of mental health symptoms. Over the 4-year period, there were not significant differences in days of mental health symptoms by problems paying medical bills or insurance status. Despite improvements in coverage, the relationship between problems paying medical bills and mental health symptoms was not modified.

## 1. Introduction

The rising costs of healthcare are a major financial concern and create substantial barriers to accessing affordable, coordinated, continuous care for many Americans [1]–[8]. The results of a Kaiser Family Foundation (KFF) survey published in 2017 found that 29% of Americans aged 18–64 (non-elderly adults) reported problems paying medical bills, and 22% skipped needed care due to the cost [3]. Research based on 2015–2017 National Health Interview Survey data also indicate that 28.9% of Americans aged 18-64 reported financial hardship from medical bills compared to 15.3% of those aged ≥ 65 years [9]. Consistent with data from earlier studies [5]–[7], the 2017 KFF survey also showed that individuals who were uninsured (41%), low income (42%) or in poor/fair heath (52%) reported more problems paying medical bills than insured (30%), higher income (13%), healthier individuals (23%) [3].

Difficulties paying medical bills and the related problem of accumulating medical debt, have remained at the forefront of public policy debates in some states [10], including prescription drug affordability [11]. A primary goal of the Affordable Care Act (ACA) was to make affordable health insurance available to more people and, consequently, to ensure access to essential health benefits and alleviate the financial burden of medical care [4],[12],[13]. The ACA has resulted in more non-elderly Americans being insured and gaining access to care. However, a greater number of people in this group are now underinsured and experience high out-of-pocket costs and deductibles [2]. Exposure to such expenses has not only resulted in reported problems paying bills, but also increased medical debt [2], further underscoring worries about affordability.

Medical bills and debt can take a serious toll on mental well-being. A systematic review of the literature showed that individuals with debt are three times as likely to have a mental health problem [14], such as anxiety, stress, or depression [15]–[17]. In the 2017 KFF survey of non-elderly adults, 25% of the insured and 39% of the uninsured were “very worried” about not being able to afford needed healthcare services [3]. Another 2017 study by the American Psychological Association found that three in five Americans were stressed about medical bills (57%) and the cost of medications (60%) [15]. Additionally, worrying about debt has been associated with mental health issues [18], which may result in depression and other acute or chronic mental health problems [16]. Stressors including cost of health insurance and being able to obtain adequate, or “good” health insurance may also trigger mental health symptoms [15],[19]. For example, Jacobs et al. [19] in a study of low-income women found that having public insurance coverage was unrelated to depression but associated with greater anxiety.

While healthcare coverage has substantially improved for low-income individuals and populations eligible for expanded Medicaid coverage [20], ACA coverage gains have not translated into reductions in problems paying medical bills for all [2]. Mental well-being may also not have changed for all populations. In theory, ACA coverage gains would reduce medical bill problems and consequently mental well-being. However, research shows that the extent to which insurance coverage gains have improved health is equivocal [20]–[22]. It is unclear whether ACA coverage expansions and improvements have correlated with improved well-being. Given the increasing burden of patient cost-sharing [2], and the vicious cycle of financial distress and poor mental health [23], it is critical that we understand mental well-being within context of the ACA achievements. In the present analysis, we addressed the following research question: Did mental health symptoms decrease with the improved ability to pay medical bills?

Guided by the Andersen's Behavioral Model of Health Services Use (BMHSU) [24],[25], we examine whether the relationship between problems paying medical bills and mental health symptoms changed among non-elderly adults following the implementation of the ACA. The BMHSU, one of the most widely used models to explain health services use, posits that predisposing, enabling, and need factors influence health services use and outcomes [24],[25]. The relationships among these factors and outcomes are also reciprocal. Medical bill problems can be viewed as an intermediate outcome and an important enabling factor of health services use [26]–[28]. Our previous work has established the value of the BMHSU in understanding predictors, mediators, and outcomes of medical bill problems [5],[29],[30].

## 2. Methods

### 2.1. Data source and sample

Data for our analyses were drawn from the Health Reform Monitoring Survey (HRMS) for the years 2013 to 2016. Developed with the purpose of providing early data regarding the implementation issues of the Affordable Care Act (ACA) [31],[32], the HRMS tracks information on health insurance coverage, access to care, affordability of care, and health status. Each round of the HRMS is conducted in a random sample of approximately 7,500 individuals, who are drawn from probability-based, nationally representative internet panel of 55,000 civilian, non-institutionalized Americans ages 18–64. HRMS data were collected quarterly from 2013–2014 and biannually beginning in 2015. For this study, we pooled data from quarterly rounds to be consistent with the biannual rounds; thus, unweighted sample sizes for each biannual period ranged from 8,253 to 16,128 individuals. Our analytic samples ranged from 7,197 to 15,356 individuals. Missing values were not imputed, but item non-response rates for HRMS are generally low (less than three percent). The percentage missing in our analysis for the outcome and key independent variables were as follows: 1.3% for days of mental health symptoms, 0.46% for problems paying medical bills, and 2.2% for insurance status. Our analysis omitted 4.4% of the data from 2013–2016 waves of HRMS. The HRMS receives its core funding from the Robert Wood Johnson Foundation and Urban Institute (http://hrms.urban.org/about.html). Detailed descriptions of the HRMS and documentation are available from the Health and Medical Care Archives (http://www.icpsr.umich.edu/HMCA/) and have been published elsewhere [31]–[33].

### 2.2. Dependent variable

The dependent variable of interest is a continuous measure assessing the number of days respondents experienced stress, depression, or problems with emotions in the past 30 days. The HRMS used the following question to assess mental health symptom days: *Now thinking about your mental health, which includes stress, depression, and problems with emotions, for how many days during the past 30 days was your mental health not good*? The responses ranged from 0 to 30 days.

### 2.3. Independent variables

The Andersen Behavioral Model of Health Services Use guided our selection of independent variables [24]. The predisposing factors in our analysis included mutually exclusive categories for race/ethnicity (White non-Hispanic, Black non-Hispanic, other non-Hispanic, and Hispanic), age (18–34, 35–49, 50–64), gender (male/female), education level (less than high school, high school, some college, Bachelor's degree or higher), and usual source of care (yes/no). The enabling factors included categories representing income as a percent of poverty level, which are based on the U.S. Department of Health and Human Services annual poverty guidelines and reflect ACA premium subsidy eligibility (≤ 138% of the federal poverty level (FPL), 139–399% of the FPL, and ≥400% of the FPL); insurance status (insured/ uninsured); and problems paying medical bills (yes/no). The following HRMS question was used to assess affordability of care: *In the past 12 months did you or anyone in your family have problems paying or were unable to pay any medical bills*? Responses were coded yes or no. Insurance status and problems paying medical bills were the main independent variables of interest in our study. The “need” factor was assessed with perceived health status (poor/fair, good, very good, excellent).

Additionally, we included a variable to account for the effect of the eight distinct biannual survey periods over time (time 1 [2013 January-June] to time 8 [2016 July-December]).

## 3. Analysis

We performed a descriptive analysis (frequencies or means) on study variables for each biannual period, and assessed the changes in prevalence of mental health symptom days (i.e., the days having stress, depression, and problems with emotions during the past 30 days) by problems paying medical bills from 2013 to 2016. The outcome variable (i.e., mental health symptom days) contained an excess number of zero values (approximately 60%)—indicating a zero-inflated model was required to fit the data. Zero-inflated models are two-part models: one part for predicting the probability of excess zeros and the other for assessing the number (or count) of mental health symptom days [34]–[36]. We evaluated the mean and variance for overdispersion of the count responses to determine the most appropriate model (zero-inflated Poisson (ZIP) compared to zero-inflated negative binomial (ZINB)) for the data [34]–[36]. The variance (92.44) was greater than the mean (9.75) for count days of mental health symptoms, which is an indication of overdispersion [34],[36]. The ZINB model, which accounts for excess zeros and over-dispersion, was selected to assess the relationship between problems paying medical bills and mental health symptom days, and changes in this relationship over time. The over-dispersion parameter alpha (α = 1.350; 95% CI: 1.308–1.393) and the likelihood ratio-test (p < 0.001) were significant confirming that the ZINB model was a better fit for the data than the ZIP model [34]–[36]. In the ZINB model, we also examined the interaction between insurance and problems paying medical. Betas and standard errors are used to assess the strength of association. A p-value < 0.05 was considered significant for all statistical tests. For all analyses, we accounted for the complex sampling design of the HRMS [31]–[33]. All analyses were performed using with STATA software, version 13.1 (StataCorp, LP; College Station, Texas).

## 4. Results

Table 1 shows the characteristics of respondents by initial and final year. The percentage of uninsured decreased over the 4-year period from 15.1% to 9.0% and the percentage of those who reported problems paying medical bills decreased from 22.0% to 18.6%. The percentage of respondents reporting one or more days of mental health symptoms increased from 39.3% to 42.9%. Overall, the majority of the uninsured tended to be young (18–34 years) and in very good or excellent health (results not shown). In 2013, the youngest age group had the highest percentage (23.2%) of problems paying medical bills, compared to those aged 35–49 years (21.6%) and 50–64 years (20.9%). From 2013 to 2016, problems paying medical bills decreased by 4.8% for 18–34 year olds compared to 1.8% for those aged 35–49 years and 3.0% for those aged 50–64 years.

**Table 1.** Sample characteristics by initial and final study period and by problems paying medical bills (weighted %, unweighted n): health reform monitoring survey, united states, 2013–2016.

	2013^a^ (n = 10,152	2016^b^ (n = 7,912)	Unable to Pay Medical Bills in the Past 12 Months	
			2013^a^ (n = 2,100)	2016^b^ (n = 1,515)
Age				
18–34	36.0 (2,613)	35.7 (2,138)	23.2 (564)	18.4 (421)†
35–49	30.8 (3,006)	30.5 (2,265)	21.6 (649)	19.8 (461)
50–64	33.2 (4,533)	33.8 (3,509)	20.9 (887)	17.9 (663)†
Gender				
Female	50.9 (5,280)	51.1 (4,141)	24.4 (1,214)	21.6 (902)†
Male	49.1 (4,872)	48.9 (3,771)	19.4 (886)	15.6 (613)†
Race/ethnicity				
White	64.2 (7,446)	62.8 (5,412)	20.0 (1,412)	17.3 (964)†
Black	11.8 (854)	12.2 (709)	24.2 (217)	22.9 (167)
Hispanic	16.0 (1,222)	16.4 (1,151)	29.9 (345)	23.9 (277)†
Other	8.0 (630)	8.6 (640)	18.4 (126)	12.2 (107)†
Poverty Level				
Income ≤ 138% FPL^c^	27.4 (2,074)	27.1 (1,946)	35.0 (737)	28.9 (581)†
Income 139%–399% FPL^c^	36.5 (4.257)	36.0 (3,132)	25.0 (1,034)	21.9 (714)†
Income ≥ 400% FPL^c^	36.1 (3,820)	36.9 (2,834)	9.0 (329)	8.0 (220)
Education				
Less than high school	30.7 (3,849)	31.3 (2,509 )	32.3 (244)	24.6 (196)†
High school	30.3 (3,129)	29.9 (2,601)	27.2 (626)	21.5 (446)†
Some college	28.3 (2,407)	28.0 (2,061)	23.2 (733)	21.3 (572)
Bachelor or higher	10.7 (767)	10.8 (741)	12.2 (498)	11.5 (301)
Usual Source of Care				
No	30.0 (2,834)	26.0 (1,941)*	22.1 (595)	19.1 (388)†
Yes	70.0 (7,318	74.0 (5,971)	21.0 (1,505)	18.5 (1,127)†
Health Status				
Poor/fair	13.1 (1,331)	14.0 (1,209)	39.5 (523)	35.2 (424)
Good	35.2 (3,613)	34.7 (2,802)	25.8 (881)	21.3 (599)†
Very good	38.6 (3,957)	39.4 (3,027)	15.3 (542)	12.3 (390)†
Excellent	13.0 (1,251)	11.9 (874)	13.7 (154)	12.4 (102)
Insurance				
No	15.1 (1,299)	9.0 (647)*	35.7 (453)	28.5 (197)†
Yes	84.9 (8,853)	91.0 (7,265)	19.5 (1,747)	17.7 (1,318)†
Days of Mental Health Symptoms				
Zero days	60.7 (6,238)	57.1 (4,517)*	15.3 (905)	12.4 (573)†
One or more days	39.3 (3,914)	42.9 (3,395)	32.2 (1,195)	26.9 (942)†
Problems Paying Medical Bills				
Unable to pay	22.0 (2,100)	18.6 (1,515)*		
Able to pay	78.0 (8,052)	81.4 (6,397)		

^*^Notes: ^a^ January-June (initial study period is 2013^a^); ^b^ July-December (final study period is 2016^b^), There are 6 study periods between 2013 and 2016; ^c^ Federal Poverty Level; † *p* < 0.05 for comparisons between 2013^a^ and 2016^b^ on having problems paying medical bills; * *p* < 0.05 for association between initial and final study period (year) and respective variable.

**Figure 1.**
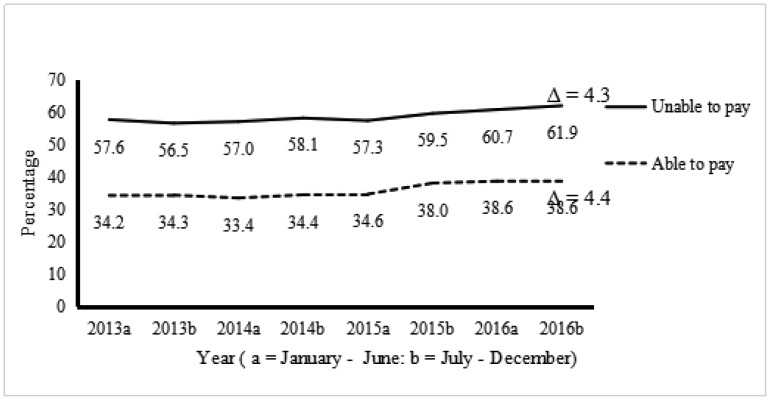
Prevalence of >1 Days of Mental Health Symptoms by Able to Pay and Unable to Pay Medical Bills: HRMS: U.S., 2013–2016.

Figure 1 shows the prevalence of having one or more mental health symptom days by reported problems paying medical bills for each biannual period from 2013–2016. The prevalence of one or more days of mental health symptoms increased for respondents who had problems paying medical bills (57.6% to 61.9%, ?PR = 4.3%, 95% CI: 0.6% to 7.9%), as well as those who did not have problems paying (34.2% to 38.6%, ?PR = 4.4, 95% CI: 2.5% to 6.2). Overall, the average days of mental health symptoms were higher for respondents who had problems paying medical bills but increased for both those with and without problems paying medical bills (6.6 to 7.5 versus 3.0 to 3.6, respectively; results not shown).

Table 2 shows regression coefficients and standard errors from the ZINB model. The logit portion (i.e., the zero count group or individuals who reported no days of mental health symptoms), indicate that individuals who had problems paying medical bills had a lower probability of reporting zero days of mental health symptoms (Beta = −0.845, p < 0.001), compared to those who did not have problems paying medical bills. The uninsured, compared to the insured, had a higher probability of reporting zero days of mental health symptoms (Beta = 0.299, p < 0.001). There were no significant differences in the zero days of mental health symptoms based on whether respondents reported problems paying medical bills or their insurance status over time (results not shown). The interaction of problems paying medical bills and insurance status was not significant.

**Table 2.** ZINB regression coefficients for days of mental health symptoms (n = 85,430): health reform monitoring survey, U.S., 2013–2016.

	Logit Model			Negative Binomial Model		
	β	Std Err	*p-value*	β	Std Err	*p-value*
Age						
18–34 (reference)						
35–49	0.373	0.029	<0.001	0.004	0.018	0.821
50–64	0.761	0.028	<0.001	−0.007	0.018	0.663
Gender (reference: male)	0.696	0.022	<0.001	0.023	0.015	0.143
Race/ethnicity						
White (reference)						
Black	0.319	0.037	<0.001	0.070	0.025	0.006
Hispanic	0.245	0.035	<0.001	0.015	0.023	0.496
Other	0.128	0.048	0.008	−0.078	0.034	0.021
Poverty level						
Income ≤ 138% FPL^c^	0.179	0.030	<0.001	−0.143	0.017	<0.001
Income 139%–399% FPL	0.376	0.035	<0.001	−0.206	0.023	<0.001
Income ≥ 400% FPL (reference)						
Education						
Less than high school	0.220	0.027	<0.001	0.239	0.020	<0.001
High school	0.493	0.030	<0.001	0.229	0.022	<0.001
Some college	0.331	0.048	<0.001	0.239	0.030	<0.001
Bachelor or higher (reference)						
Have usual source of care	0.196	0.026	<0.001	−0.003	0.017	0.844
Health Status						
Poor/fair	−1.912	0.048	<0.001	0.727	0.037	<0.001
Good	−0.993	0.039	<0.001	0.292	0.036	<0.001
Very good	−0.519	0.038	<0.001	0.037	0.037	0.320
Excellent (reference)						
Uninsured	0.299	0.039	<0.001	0.020	0.025	0.414
Unable to Pay Medical Bills	−0.845	0.031	<0.001	0.131	0.016	<0.001
Time (bi-annually)						
January-June, 2013 (reference)						
July-December, 2013	−0.019	0.041	0.963	0.025	0.028	0.359
January-June, 2014	0.035	0.041	0.401	−0.024	0.028	0.392
July-December, 2014	0.013	0.041	0.748	0.013	0.028	0.649
January-June, 2015	0.025	0.048	0.603	0.021	0.033	0.573
July-December, 2015	−0.151	0.047	0.001	−0.006	0.032	0.861
January-June, 2016	−0.143	0.047	0.002	0.092	0.031	0.003
July-December, 2016	−0.182	0.046	<0.001	0.063	0.031	0.040

^*^Notes: ^c^ Federal poverty level.

The negative binomial portion (individuals reporting 1 to 30 days of mental health symptoms) indicate that individuals who had problems paying medical bills had more count days of mental health symptoms (Beta = 0.133, p < 0.001) than those who did not have problems paying medical bills. Insurance was not a significant predictor of count days of mental health symptoms. Over the 4-year period, there were no statistically significant differences in the count days of mental health symptoms by ability to pay medical bills or insurance status (results not shown). There were no significant interactions by ability to pay medical bills and insurance status.

## 5. Discussion

The major findings of this study are that Americans with problems paying medical bills had more days of mental health symptoms than those who did not have problems paying medical bills; however, from 2013 to 2016, there were no significant differences in days of mental health symptoms by problems paying medical bills. Nonetheless, differences in days of mental health symptoms between those unable to pay (∼6.5 days) and able to pay (∼3.2 days) were large and increased slightly at similar rates over time. Overall, insurance coverage and problems paying medical bills improved over the 4-year period, but the prevalence of mental health symptoms worsened.

Consistent with that of other studies on financial distress and mental health [14],[37], we found more days of mental health symptoms among those with problems paying medical bills. While there were no statistically significant differences over time, recent studies have shown high or increasing levels of stress, anxiety, or depression among Americans regarding their ability to pay medical bills [9],[15],[38]–[40]. These studies assessed stress, anxiety, and depression as individual measures, unlike our study that used a global measure of mental health symptoms. With increasing health care costs and higher patient cost-sharing [1],[2],[41]–[43], mental health symptoms will likely be higher among individuals with problems paying medical bills. Research on mental health trends in the USA indicate the increasing prevalence among vulnerable populations [44],[45].

Our findings suggest that non-elderly Americans had improved ability to pay medical bills but worsening mental health symptoms. On first consideration, this finding seems paradoxical; however, it is not inconsistent with findings from previous research on health insurance expansions [46]. For example, some studies show that improve affordability from Massachusetts reform was associated with improved health status [47],[48]. On the other hand, Yelowitz and Cannons [20] concluded from their analysis of the Massachusetts reform, that the law was more successful in providing insurance coverage than changing population health. There is also ambiguity in the literature about the extent to which ACA insurance coverage gains have improved health [21],[22]. Our study's novelty and contribution was to assess and clarify how the ACA's impact on the ability to pay medical bills correlated with mental health symptoms over time.

Improved insurance coverage and affordability does not translate to improvement in health when health care systems are fragmented and inefficient [43]. There is ample evidence indicating that Americans are persistently concerned about healthcare costs and access issues [38],[39], and are uncertain about the changing healthcare system [15],[49]. In the past decade, availability and affordability of healthcare consistently top the list of Americans' concern [38],[39].Consequently, our finding of improved ability and increasing prevalence of mental health symptoms overtime may be capturing worries about health care issues.

Of note, the uninsured were significantly more likely to report having zero days of mental health symptoms than the insured. However, conditional on having one or more days of mental health symptoms, insurance was not a significant predictor of the count days of mental health symptoms. This relationship may reflect the connection between health status and the need for health services [24]. An exploration of the HRMS data showed that the majority of the uninsured tended to be young (18–34 years) and in very good or excellent health. Consequently, they are less likely to use medical care and experience problems from medical bills [50].

There are some limitations that are worth noting in this study. First, the data is self-reported which is subject to recall and measurement biases. Second, the internet-based survey may result in some groups (e.g., low-income and undereducated) being underrepresented due to inequitable access to the internet and computers. Third, the data were cross-sectional, making causality hard to establish. Fourth, the outcome measure did not directly assess mental health symptoms pertaining to problems paying medical bills. Nonetheless, stress, anxiety, and depression as measured by our outcome variable, are among the most common mental health symptoms that people experience related to problems paying bills and increased debt [14],[15],[40]. Finally, it is possible that there is a bidirectional relationship between mental health symptoms (i.e., stress, anxiety, and depression) and problems paying medical bills, that is, more days of mental health symptoms may lead to medical bills problems and vice versa. Establishing this bidirectional link would require contextual factors including timing and duration of medical bill problems and mental health symptoms. Finally, the analysis does not account for whether respondents were in Medicaid expansion or non-expansion states. Courtemanche and colleagues [21] found that ACA private insurance expansion accounted for improvements in health rather than Medicaid expansion.

### 5.1. Policy implications

Our findings have implications for improving mental health and affordability issues post ACA implementation. Options or strategies for improvement include exploring effective insurance designs, innovative models of care, and consumer engagement models [2],[51],[52]. According to research conducted by the Commonwealth Fund [51], comparing cost, access, and affordability in the U.S. system and 10 other industrialized countries, addressing the issues in the U.S. system will necessitate balancing patients' cost-sharing with their ability to pay, providing regulatory support to increase access to primary care, and addressing the administrative burden and complexity of insurance coverage. Accountable Care Organizations (ACOs) (i.e., doctors, hospitals and other health care providers form networks to coordinate patient care) are proposed as a solution to achieve higher quality, lower cost, and improved population health, but the results on these organizations have been mixed [53]. Patient engagement, such as cost of care conversations between patients and providers, has been shown to lower overall healthcare and patients' costs [54]–[56]. However, such conversations often do not take place or are done in a disorganized manner [54]. Tools for these conversations require further development or study [57].

## 6. Conclusions

Problems paying medical bills contribute to financial and emotional distress for many Americans, even the insured. This study demonstrates that despite significant gains in health insurance coverage and improved ability to pay medical bills post ACA implementation, patterns in mental health symptoms have remained largely unchanged among non-elderly adults in the United States. Policies that balance availability, affordability, and coverage of effective care may mitigate the mental health symptoms that Americans experience from difficulty paying medical bills. Single-payer systems compared to multi-payer systems like the U.S., have been shown to be more successful in providing coverage and financial protections for individuals, ensuring effective care and outcomes, while controlling costs [51],[58],[59]. Reforms to further expand Medicaid, enhance coverage in the ACA insurance marketplaces, and regulate employer-based insurance coverage to protect workers can move the U.S. multi-payer system to a more single-payer type system [58].
